# Polymer lipid hybrid nanoparticles for phytochemical delivery: challenges, progress, and future prospects

**DOI:** 10.3762/bjnano.15.118

**Published:** 2024-11-22

**Authors:** Iqra Rahat, Pooja Yadav, Aditi Singhal, Mohammad Fareed, Jaganathan Raja Purushothaman, Mohammed Aslam, Raju Balaji, Sonali Patil-Shinde, Md Rizwanullah

**Affiliations:** 1 Department of Pharmaceutical Technology, Meerut Institute of Engineering and Technology, Meerut-250005, Uttar Pradesh, India; 2 Department of Basic Medical Sciences, College of Medicine, AlMaarefa University, P.O. Box 71666, Riyadh 11597, Saudi Arabia; 3 Department of Orthopaedics, Saveetha Medical College and Hospital, Saveetha Institute of Medical and Technical Sciences (SIMATS), Saveetha University, Chennai-602105, Tamil Nadu, India; 4 Pharmacy Department, Tishk International University, Erbil 44001, Kurdistan Region, Iraq; 5 Center for Global Health Research, Saveetha Medical College and Hospital, Saveetha Institute of Medical and Technical Sciences (SIMATS), Saveetha University, Chennai-602105, Tamil Nadu, India; 6 Department of Pharmaceutical Chemistry, Dr. D.Y Patil Institute of Pharmaceutical Sciences and Research, Pimpri Pune-411018, Maharashtra, India; 7 Centre for Research Impact & Outcome, Chitkara College of Pharmacy, Chitkara University, Rajpura 140401, Punjab, India

**Keywords:** bioavailability, phytochemical, polymer lipid hybrid nanoparticles, solubility, stability, surface modification

## Abstract

Phytochemicals, naturally occurring compounds in plants, possess a wide range of therapeutic properties, including antioxidant, anti-inflammatory, anticancer, and antimicrobial activities. However, their clinical application is often hindered by poor water solubility, low bioavailability, rapid metabolism, and instability under physiological conditions. Polymer lipid hybrid nanoparticles (PLHNPs) have emerged as a novel delivery system that combines the advantages of both polymeric and lipid-based nanoparticles to overcome these challenges. This review explores the potential of PLHNPs to enhance the delivery and efficacy of phytochemicals for biomedical applications. We discuss the obstacles in the conventional delivery of phytochemicals, the fundamental architecture of PLHNPs, and the types of PLHNPs, highlighting their ability to improve encapsulation efficiency, stability, and controlled release of the encapsulated phytochemicals. In addition, the surface modification strategies to improve overall therapeutic efficacy by site-specific delivery of encapsulated phytochemicals are also discussed. Furthermore, we extensively discuss the preclinical studies on phytochemical encapsulated PLHNPs for the management of different diseases. Additionally, we explore the challenges ahead and prospects of PLHNPs regarding their widespread use in clinical settings. Overall, PLHNPs hold strong potential for the effective delivery of phytochemicals for biomedical applications. As per the findings from pre-clinical studies, this may offer a promising strategy for managing various diseases.

## Introduction

Phytochemicals are naturally occurring compounds found in plants (from the Greek word “phyton” meaning plant) [1]. These bioactive compounds are responsible for the color, flavor, and aroma of plants, but more importantly, they contribute to the plant’s defense mechanisms against pathogens, pests, and environmental stresses. Beyond their role in plant biology, phytochemicals have gained significant attention for their potential health benefits in humans [2–3]. Although they are not essential nutrients like vitamins and minerals, phytochemicals play a crucial role in maintaining health and preventing disease. These compounds are broadly categorized into several classes, including alkaloids, flavonoids, phenolic acids, terpenoids, and glycosides, each with distinct chemical structures and biological activities [4–5].

Phytochemicals have captured the interest of the scientific community and the pharmaceutical industry alike because of their extensive therapeutic potential. They function as antioxidants, anti-inflammatory agents, anticancer compounds, and antimicrobials, offering a natural and multifaceted arsenal for combating a wide array of diseases [6–8]. Despite their promising bioactivities, the clinical application of phytochemicals is often limited by several inherent drawbacks such as poor water solubility, low bioavailability, rapid metabolism, and instability under physiological conditions [9–11]. These challenges necessitate the development of advanced delivery systems to harness the full potential of phytochemicals in therapeutic applications.

Polymer lipid hybrid nanoparticles (PLHNPs) represent an innovative class of delivery vehicles that combines the beneficial properties of both polymeric and lipid-based nanoparticles, thus offering a creative approach to enhance the delivery and efficacy of drugs/phytochemicals. The architecture of PLHNPs typically consists of a lipid core or shell enveloped by a polymer matrix, which can vary in its configuration depending on the specific requirements of the delivery system [12–13]. The lipid components, often phospholipids, cholesterol, and surfactants are integral for solubilizing lipophilic drugs. The polymer component, which can include materials such as polyethylene glycol (PEG), poly(lactic-*co*-glycolic acid) (PLGA), polycaprolactone (PCL), and chitosan (CHS), provides structural integrity, controlled release properties, and protection against premature degradation [14–15]. This hybrid structure improves the encapsulation efficiency of phytochemicals/drugs and enhances their solubility, stability, and bioavailability. Additionally, the physicochemical characteristics of PLHNPs can be tailored by varying the concentrations of polymers and lipids [16].

PLHNPs can address various challenges associated with phytochemical delivery. The benefits of PLHNPs include their small particle size, high encapsulation efficiency, enhanced stability, and improved dissolution in harsh gastrointestinal (GI) fluids. Following oral administration, PLHNPs demonstrate superior intestinal absorption and bioavailability, attributed to their enhanced stability and dissolution rate in GI fluids. Further, PLHNPs overcome limitations such as rapid metabolism and limited bioavailability by encapsulating phytochemicals within the hybrid matrices. This encapsulation enhances the stability of phytochemicals, extends their circulation time within the body, and enhances their therapeutic effectiveness [17–18]. Additionally, PLHNPs possess the capability to encapsulate and co-deliver two drugs with distinct physicochemical characteristics to a targeted site and show synergistic therapeutic efficacy. In PLHNPs, the lipophilic drugs are encapsulated within the polymeric core, while hydrophilic drugs are entrapped in the lipid shell. PLHNPs demonstrate relatively greater loading capacity for lipophilic compounds than other nanoparticle systems [12,19].

Moreover, the surface modification of PLHNPs with targeting ligands, such as antibodies, peptides, or aptamers, has been explored to improve the selective delivery of drugs/phytochemicals to specific tissues or cells. A site-specific targeting approach enhances the therapeutic efficacy of phytochemicals and reduces systemic toxicity. In addition to enhancing solubility and targeting, PLHNPs offer controlled release properties that are crucial for maintaining therapeutic drug levels over extended periods of time [20–21]. By adjusting the polymer composition and lipid matrix, researchers can fine-tune the release kinetics of phytochemicals, ensuring sustained therapeutic effects. This controlled release mechanism is particularly advantageous for chronic conditions requiring long-term treatment, such as cancer, cardiovascular diseases, and neurodegenerative disorders [22–23].

This review aims to discuss the ability of PLHNPs to improve the therapeutic delivery of phytochemicals for biomedical applications. In this review, we discuss the obstacles in the conventional delivery of phytochemicals, types of PLHNPs, different phytochemical-loaded PLHNPs for improved phytochemical delivery, challenges in clinical translation of PLHNPs, and future perspectives.

## Review

### Obstacles in the conventional delivery of phytochemicals

The conventional delivery of phytochemicals faces numerous challenges that limit its clinical application and therapeutic efficacy. These challenges arise from the physicochemical characteristics of phytochemicals, as well as from the physiological barriers they encounter in the body. One major challenge is poor water solubility. Many phytochemicals are hydrophobic and show poor water solubility, which significantly restricts their absorption in the gastrointestinal tract (GIT) when administered orally. This low solubility leads to low bioavailability, resulting in sub-therapeutic levels of the phytochemical at the target site [24–25]. Another significant issue is low bioavailability. Bioavailability refers to the proportion of an administered substance that enters the bloodstream and is available for therapeutic action. Phytochemicals often exhibit low bioavailability due to poor solubility, rapid metabolism, and degradation in the physiological fluids. This necessitates higher doses to achieve therapeutic effects, which may increase the risk of side effects and toxicity [26–27]. Chemical instability is also a critical challenge. Phytochemicals can be chemically unstable and degrade under physiological conditions, such as varying pH levels, temperature, and enzymatic activity. Degradation reduces the effective concentration of the phytochemical, diminishing its therapeutic potential [28–29]. Rapid metabolism and clearance further complicate phytochemical delivery. Phytochemicals are often rapidly metabolized by liver enzymes and cleared from the body through renal or biliary excretion, resulting in short plasma half-lives and requiring frequent dosing to maintain effective therapeutic levels. This rapid clearance reduces the duration of action, making it challenging to achieve sustained therapeutic effects [30–31]. Poor permeability and penetration are additional obstacles. Phytochemicals may have difficulties crossing biological membranes, such as the intestinal epithelium or the blood–brain barrier, because of their molecular size, polarity, or lipophilicity. Poor permeability limits the ability of phytochemicals to reach intracellular or central nervous system targets, reducing therapeutic efficacy in tissues that are difficult to access. Variability in absorption is another significant issue. The absorption of phytochemicals can be influenced by various factors, including food intake, gut microbiota, and individual genetic differences. This variability leads to inconsistent therapeutic outcomes among different individuals, and food–drug interactions can further complicate dosing regimens and efficacy [32–33]. Further, conventional delivery methods often lack specificity, resulting in the distribution of phytochemicals throughout the body rather than targeting specific tissues or cells. Non-specific distribution increases the risk of off-target effects and systemic toxicity, reducing the concentration of the phytochemical at the desired site of action and decreasing therapeutic effectiveness [34–35]. In addition, conventional delivery systems often cannot provide controlled or sustained release of phytochemicals, leading to fluctuating plasma levels. These fluctuations can result in suboptimal therapeutic effects and increased side effects. Lack of controlled release is particularly problematic for chronic conditions that require consistent drug exposure over extended periods of time [36–37]. Therefore, there is a need for innovative delivery systems that improve solubility, stability, bioavailability, and targeted delivery, while also providing controlled release. PLHNPs represent a promising solution to many of these challenges, offering a versatile platform to maximize the therapeutic potential of phytochemicals.

### Overview of PLHNPs for phytochemical delivery

PLHNPs represent an innovative solution for the effective delivery of phytochemicals by harnessing the combined advantages of both polymeric and lipidic nanoparticles. PLHNPs represent higher encapsulation efficiency, ensuring that a higher proportion of the phytochemical payload is successfully encapsulated within the hybrid matrix of nanoparticles [38–39]. This improved encapsulation leads to enhanced bioavailability, meaning more of the phytochemical can reach the systemic circulation, resulting in greater therapeutic efficacy. Structurally, PLHNPs maintain their integrity through a unique hybrid architecture. They typically consist of a lipid core or shell surrounded by a polymer matrix. The lipid components, including phospholipids, cholesterol, and surfactants, play a crucial role in solubilizing lipophilic phytochemicals and facilitating interactions with biological membranes. The polymers provide structural stability, controlled release properties, and protection against premature degradation [40–41]. PLHNPs address various challenges associated with phytochemical delivery. They overcome limitations such as poor solubility, rapid metabolism, and limited bioavailability by encapsulating phytochemicals within lipid–polymer hybrid matrices. This encapsulation enhances the stability of phytochemicals, prolongs their circulation time in the body, and enhances their therapeutic effectiveness [42–43]. Additionally, surface engineering of PLHNPs with different ligands facilitates specific delivery of drug/phytochemicals to desired tissues or cells, reduces their adverse effects, and improves their therapeutic efficacy [44]. The development of PLHNPs for phytochemical delivery holds significant promise across various biomedical applications. PLHNPs can be utilized in cancer therapy, cardiovascular disease management, neurodegenerative disorder treatment, and other areas of medicine where phytochemicals show therapeutic potential.

### Types of PLHNPs

**Figure 1:**
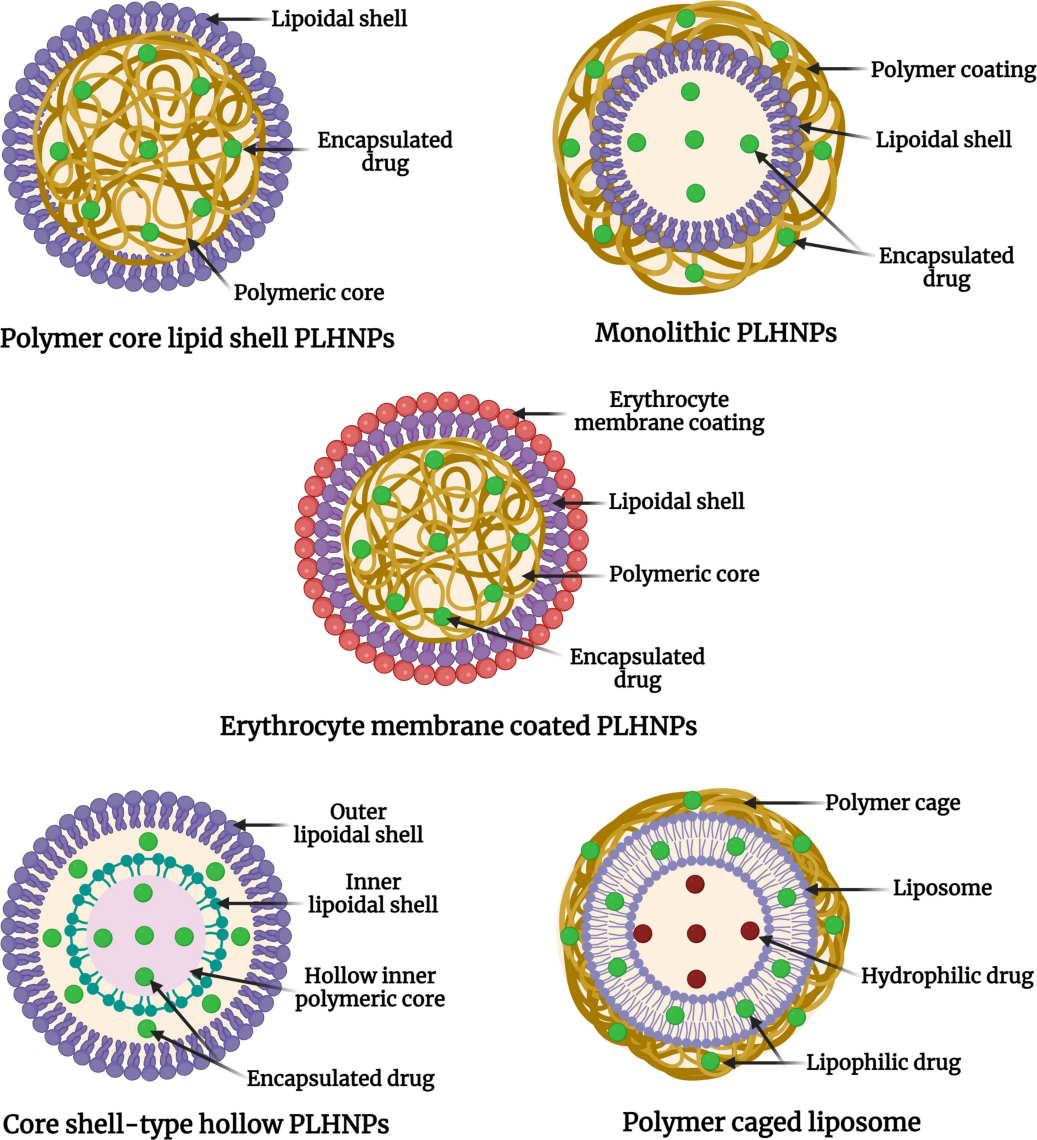
Different types of PLHNPs. Figure 1 was created in BioRender. Rizwanullah, M. (2024) BioRender.com/c14a571. This content is not subject to CC BY 4.0.

Generally, PLHNPs are classified based on the arrangement of polymers and lipids within the hybrid system. In the hybrid structures, polymers enhance overall particle stability and modulate the release of encapsulated drugs from the hybrid matrix. However, lipids provide more space for drug encapsulation and biocompatibility of the system. Therefore, the advancement in the LPHNPs yields better and prolonged therapeutic efficacy. Different types and structural advantages of PLHNPs are illustrated in Figure 1 and are discussed in detail as follows.

#### Polymer core–lipid shell hybrid nanoparticles

As the name suggests, polymer core–lipid shell hybrid nanoparticles are composed of a polymer core that is covered by mono/bilayers of a lipoidal shell. The polymeric core significantly enhances the stability of the outer lipoidal shell. The biodegradable polymeric core with a stable outer lipoidal shell makes these PLHNPs an excellent nanocarrier for therapeutic drug delivery and the treatment of various diseases. The amphiphilicity of biodegradable polymers and lipids promotes the encapsulation of both lipophilic as well as hydrophilic chemotherapeutic drugs within the hybrid system. During the development of LPHNPs, different physicochemical characteristics such as size, loading capacity, charge, solubility, release, and colloidal stability can be modulated by modification in the polymer/lipid ratio [45–47].

#### Monolithic PLHNPs

Monolithic PLHNPs are the simplest among PLHNPs; they are simply mixed nanosystems of polymer/copolymer and lipids with the help of surfactants. In this system, the lipids are scattered in a polymeric/copolymeric matrix [48]. Monolithic PLHNP systems are very similar to colloidal polymeric nanocarriers. In these nanocarriers, phospholipids help to form a carrier-like structure, which is an integral part of the system. In addition, the modification of lipoidal layers with a PEG chain provides flexibility to the nanocarrier. The ratio of the polymer and lipid can easily be adjusted to modulate the physicochemical characteristics of the nanocarrier and can reduce systemic toxicity [49].

#### Core–shell type hollow PLHNPs

The core–shell type hollow PLHNPs comprise an inner hollow positively charged lipidic core, a polymeric layer in the middle, and an outer PEG lipoidal layer [50]. The inner hollow core of the system is filled with water/or buffer. Because of the positive charge, the lipids in the inner core encapsulate the drug more efficiently compared to PLHNPs with a polymeric core. In addition, because of the outer lipoidal PEG layer, these nanocarriers escape the uptake by macrophages and enhance the stability of the biological fluids [51]. During the development of these nanocarriers, the concentration of cationic lipids for the inner core, density of the PEG chain on the outer layer, and molecular weight of the polymers are adjusted to modulate their physicochemical characteristics [52–53].

#### Polymer-caged liposomes

As the name suggests, the structural arrangement of these nanocarriers involves the surface coating of liposomes with biodegradable polymers/copolymers. The surface modification not only imparts surface functionality to the nanocarrier but also enhances its therapeutic efficacy by site-specific targeting and controlled release of the encapsulated drugs. Among all PLHNPs, these hybrid nanocarriers show the highest stability in the biological fluids and stimuli-responsive release of encapsulated drugs. In addition, the polymeric cage protects the drug from the harsh environment, and encapsulated drugs can be released under specific biological conditions. Further, the polymer coating provides better colloidal stability, sustained drug release, and high loading capacity to the hybrid nanocarriers [54–56].

#### Cell membrane-camouflaged PLHNPs

PLHNPs have been coated with cell membranes (e.g., erythrocytes) to develop membrane-camouflaged PLHNPs. These hybrid nanocarriers are also called biomimetic hybrid nanocarriers because their surface chemistry mimics natural cell membranes [57]. The PLHNPs are coated with cell membranes via the extrusion technique. The coating of PLHNPs with red blood cells yields a natural vehicle for drug delivery, and these nanocarriers can easily escape the uptake by macrophages. In this system, the drugs are encapsulated in the lipophilic polymeric core, and the lipids in the outer natural membrane enhance the sustained release of drugs. With the development of these hybrid nanocarriers, the biological barriers in therapeutic drug delivery can be easily overcome. These hybrid nanocarriers show prolonged half-life and stability in biological systems, thereby enhancing therapeutic efficacy [58–59].

### Surface modification of PLHNPs

**Figure 2:**
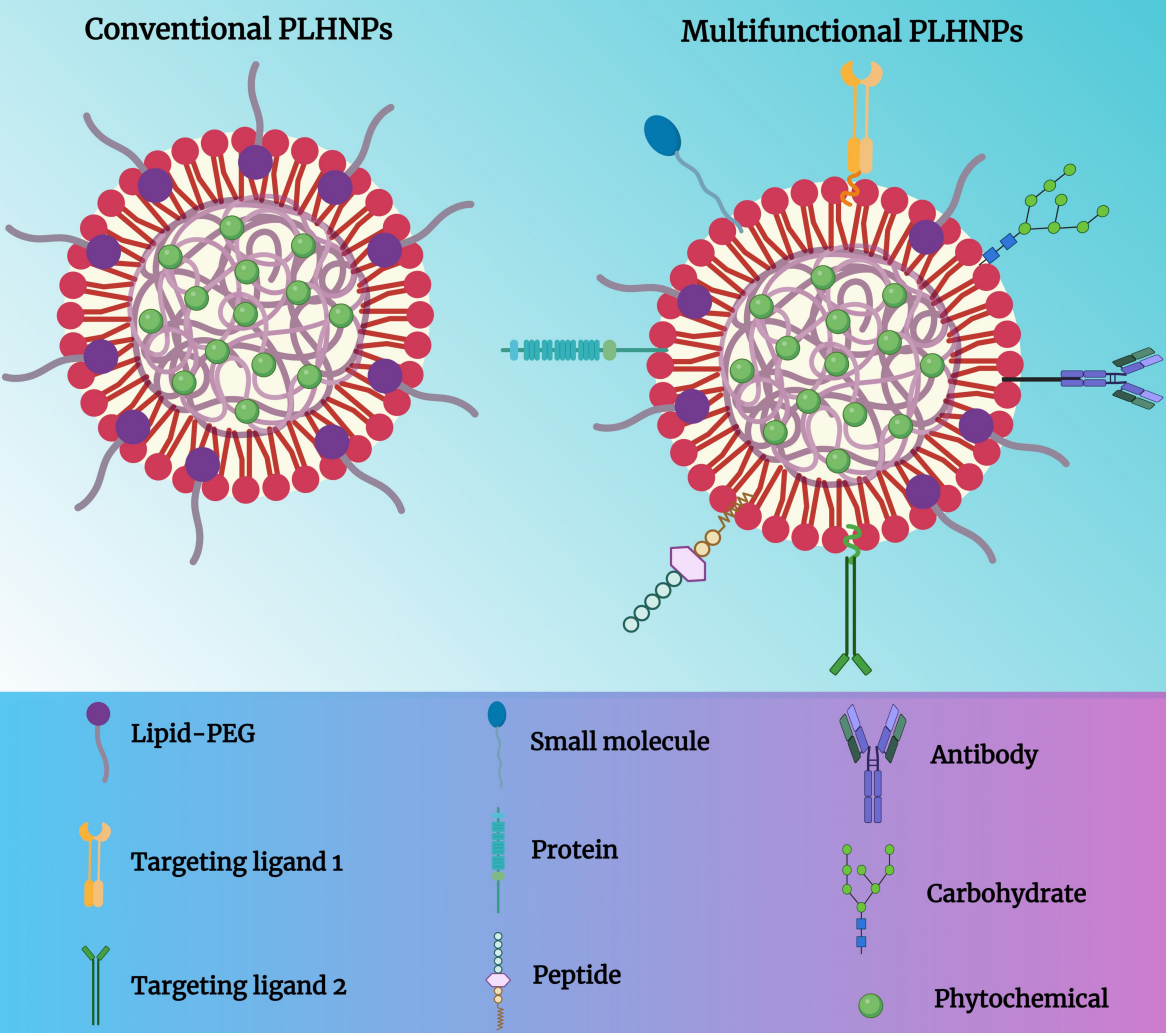
(A) Conventional PLHNPs and (B) surface-functionalized PLHNPs. Figure 2 was created in BioRender. Rizwanullah, M. (2024) BioRender.com/b56n651. This content is not subject to CC BY 4.0.

Surface modification of PLHNPs involves the functionalization of the outer surface of nanoparticles with specific molecules or ligands to impart desired properties or functionalities. This process plays a crucial role in enhancing the targeting, stability, and therapeutic efficacy of PLHNPs for drug/phytochemical delivery. Surface modification of PLHNPs begins with the preparation of the nanoparticles themselves. PLHNPs are typically synthesized using techniques such as solvent evaporation, nanoprecipitation, double emulsion, or solvent injection, resulting in the formation of nanoparticles with a lipid–polymer hybrid structure [60]. Once the PLHNPs are synthesized, their surface can be modified through various strategies. Figure 2 illustrates a comparison between conventional PLHNPs and surface-modified PLHNPs.

**Figure 3:**
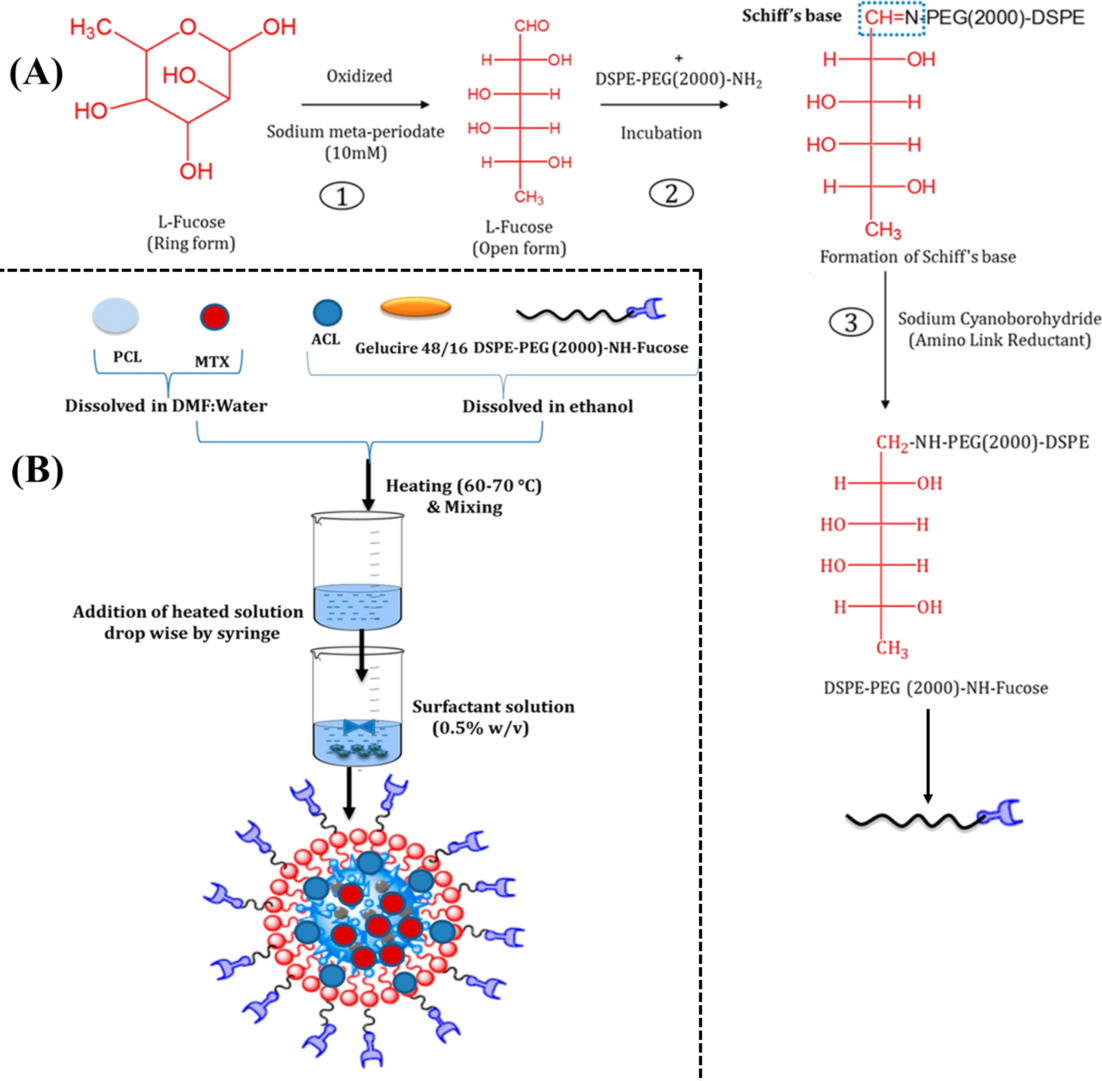
(A) Schematic illustrating the synthesis of DSPE-PEG(2000)-NH_2_-fucose; Step 1: oxidation of ʟ-fucose, step 2: formation of Schiff’s base, and step 3: reduction to a secondary amine. (B) Development of PLHNPs with fucose-decorated phospholipids. Figure 3 was reprinted with permission from [63]. Copyright 2017 American Chemical Society. This content is not subject to CC BY 4.0.

The most common approach is the attachment of targeting ligands onto the PLHNPs’ surface. These ligands can include antibodies, peptides, aptamers, or small molecules that specifically bind to receptors overexpressed on the surface of target cells or tissues. The conjugation of targeting ligands to the surface of PLHNPs enables specific delivery of drug/phytochemicals to desired sites within the body, such as tumor cells in cancer therapy. This targeted delivery can increase the therapeutic efficacy of drugs while reducing side effects by minimizing off-target effects [61–62]. For instance, Garg et al. fabricated fucose ligand-decorated PLHNPs for the co-delivery of methotrexate and aceclofenac to achieve targeted and synergistic therapeutic efficacy against breast cancer (BC) [63]. The scheme for the development of these ligand-decorated PLHNPs is depicted in Figure 3. The findings suggested that the targeted PLHNPs significantly improved uptake in BC cells by receptor-mediated endocytosis when compared with non-targeted PLHNPs. A pharmacodynamic study of targeted PLHNPs in DMBA-induced BC-bearing female BALB/c mice showed that the targeted PLHNPs yielded significantly enhanced and synergistic therapeutic efficacy and showed much better tumor inhibition and improved survival rate than the non-targeted PLHNPs.

**Figure 4:**
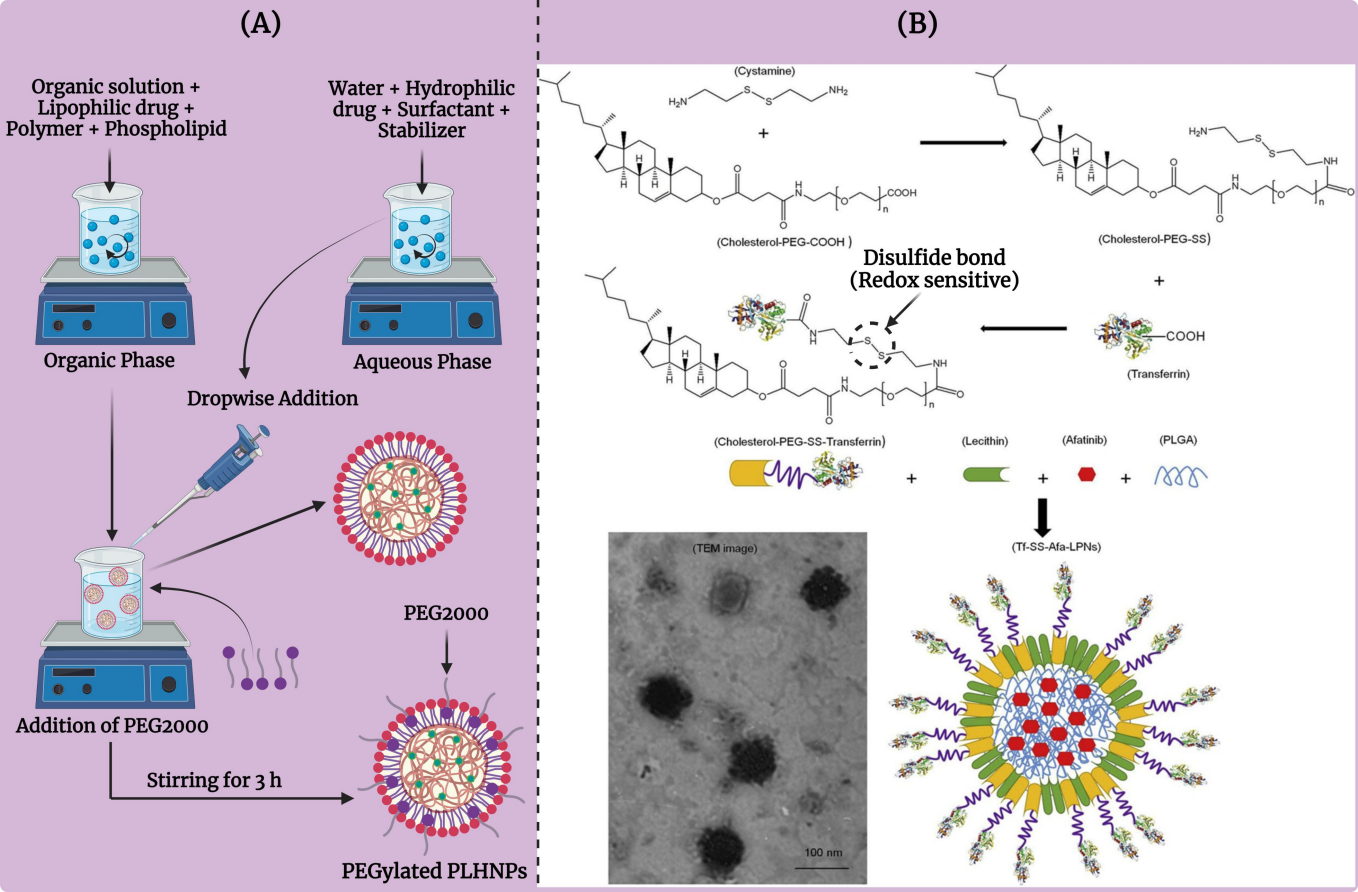
(A) Schematic illustration of the development of PEGylated PLHNPs. Figure 4A was created in BioRender. Rizwanullah, M. (2024) BioRender.com/p31e321. This content is not subject to CC BY 4.0.; (B) Scheme for the development of ligand-decorated redox-sensitive PLHNPs. Figure 4B was adapted from [75] (© 2019 J. Wang et al., published by Elsevier Masson SAS, distributed under the terms of the Creative Commons Attribution Noncommercial Noderivatives 4.0 International License, https://creativecommons.org/licenses/by-nc-nd/4.0/). This content is not subject to CC BY 4.0.

Another surface modification strategy involves the addition of stealth coatings, such as PEG, to the nanoparticle surface. A schematic representation for the development of PEGylated PLHNPs is depicted in Figure 4A. PEGylation involves attaching PEG chains to the surface of the nanoparticles. This modification provides several advantages. PEGylation increases the stability of the PLHNPs in biological fluids by preventing aggregation and reducing protein adsorption. It also extends the circulation time of nanoparticles in the bloodstream by reducing immune system recognition and clearance. This leads to improved bioavailability and allows for sustained release of the encapsulated therapeutic agents [64–66]. The advantages of PEGylated PLHNPs include enhanced biocompatibility and reduced immunogenicity. The PEG layer creates a hydrophilic barrier around the nanoparticles, which minimizes the interaction with blood components and immune cells. This reduces the risk of immune reactions and increases the half-life of the nanoparticles in the body. Additionally, PEGylation can improve the solubility of hydrophobic drugs, facilitating their delivery. The hybrid structure of these nanoparticles combines the benefits of both polymeric and lipid-based systems, offering controlled drug release and efficient encapsulation of various therapeutic agents. Overall, PEGylated PLHNPs offer a versatile and effective platform for various therapeutic applications [67–69].

Surface modification of PLHNPs can also involve the incorporation of stimuli-responsive moieties onto the nanoparticle surface. These moieties enable the nanoparticles to respond to specific stimuli, such as pH changes, temperature shifts, or enzyme activity, thereby triggering controlled drug release at the target site [70–71]. The advantages of stimuli-responsive nanoparticles include targeted and controlled drug delivery. By responding to specific stimuli, these nanoparticles can release their drug payload at the desired site of action, such as a tumor or inflamed tissue, while minimizing drug release in healthy tissues. This targeted release improves therapeutic outcomes and reduces side effects. Additionally, the ability to fine-tune the release profile based on external or internal stimuli allows for customized treatment regimens [72–73]. The hybrid structure of PLHNPs offers the combined benefits of both polymeric and lipid-based carriers, providing stability, biocompatibility, and efficient drug encapsulation. Applications of stimuli-responsive PLHNPs are vast and impactful. In cancer therapy, they are used to deliver chemotherapeutic agents specifically to tumor sites, where the acidic microenvironment or specific enzymes can trigger drug release [74]. For instance, Wang et al. fabricated transferrin ligand-decorated redox-sensitive PLHNPs to achieve improved therapeutic efficacy of afatinib against non-small cell lung cancer [70,75]. The complete scheme for the development of ligand-decorated redox-sensitive PLHNPs is represented in Figure 4B. The developed targeted PLHNPs exhibit excellent physicochemical characteristics and showed GSH-triggered drug release and significantly improved cytotoxicity against PC-9 cells in comparison to the non-targeted PLHNPs. In vivo studies suggested that the developed redox-sensitive targeted PLHNPs represented a much higher accumulation of the drug in the tumor due to targetability and higher drug release at tumor pH. Consequently, the redox-sensitive targeted PLHNPs showed much better therapeutic efficacy compared to non-targeted and non-redox-sensitive PLHNPs. Overall, surface modification of PLHNPs is a versatile strategy for enhancing nanoparticles’ targeting, stability, and therapeutic efficacy for drug/phytochemical delivery.

### PLHNPs for the delivery of single phytochemicals

**Figure 5:**
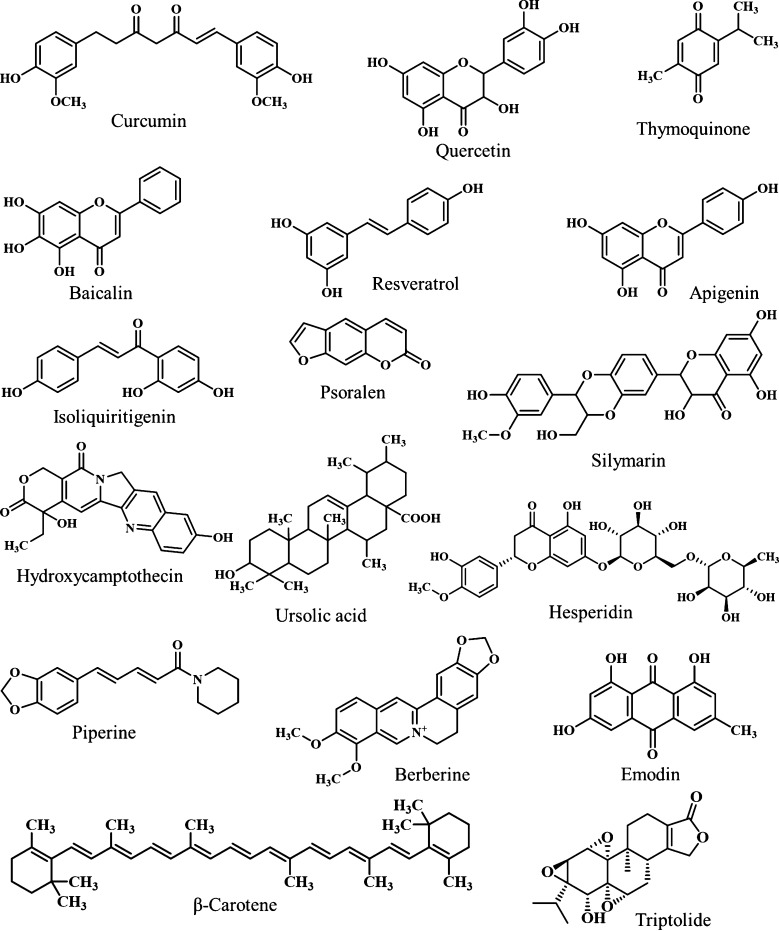
Image illustrating the chemical structures of different phytochemicals reviewed in this paper for therapeutic delivery with PLHNPs for the treatment of different diseases.

PLHNPs represent a versatile and effective platform for the delivery of single phytochemicals, offering enhanced stability, bioavailability, and targeted delivery. As previously discussed, many phytochemicals are lipophilic. Figure 5 illustrates the chemical structures of the various phytochemicals examined in this paper. After oral administration, phytochemicals exhibit poor solubility in the GI milieu and limited absorption from the GIT, resulting in low oral bioavailability and, consequently, reduced bioactivity. Recent advances regarding PLHNPs encapsulating single phytochemicals to overcome the limitations and better management of different diseases are discussed below. Pharmaceutical attributes with major outcomes are summarized in Table 1 at the end of this section.

**Table 1:** Summary of pharmaceutical properties with major findings of different phytochemical encapsulated PLHNPs.

Encapsulated Phytochemical	PLHNPs type	Pharmaceutical attributes				Ref.
	
		PS (nm)	PDI	ZP (mV)	EE (%)	

	Major outcomes					

curcumin	polymer core–lipid shell	184	–	−29.3	53.2	[82]
	Outstanding pharmaceutical attributes with excellent hemocompatibility. Notably higher cellular uptake in MCF-7 cells and a substantial decrease in the IC_50_ value compared to the free drug					

curcumin	monolithic	216.6 ± 4.7	0.205 ± 0.02	−0.23 ± 0.12	96.0 ± 0.6	[83]
	Surface modification with RGD peptide significantly improves the cellular uptake in HUVEC cells. Significantly higher anticancer therapeutic potential against B16 cells than the pure compound. Much higher in vivo antitumor activity in B16 tumor-bearing female BALB/c mice on intraperitoneal injection than the pure compound.					

quercetin	polymer core–lipid shell	339 ± 1.6	0.1 ± 0.02	−32.6 ± 0.51	78 ± 5.5	[91]
	The developed PLHNPs effectively adhere to the scalp and show significantly higher follicular uptake. Significantly higher regrowth of hair in SD rats bearing testosterone-induced alopecia.					

quercetin	monolithic	110.6	0.237	−31.9	96.22	[92]
	Enhanced cellular accumulation and antiproliferative activity against P388 cells. 3.75-fold increased bioavailability after oral ingestion in SD rats. Superior therapeutic efficacy in DBA/2 mice bearing P388 cells-induced ascitic leukemia.					

thymoquinone	monolithic	179.63 ± 4.77	0.21 ± 0.01	+26.52 ± 2.21	85.49 ± 3.73	[100]
	Outstanding mucoadhesive properties and stability in GI environments. Higher intestinal permeation and 4.74-fold increased bioavailability after oral administration compared to free compound. Markedly higher cytotoxicity against MCF-7 and MDA-MB-231 cells.					

thymoquinone	monolithic	<350	<0.3	>−19	88.42 ± 2.58	[101]
	Higher skin permeation and skin retention compared to the conventional formulation and non-irritant. Markedly enhanced antiproliferative activity against MCF-7 and MBD-MB-231 cells compared to the native compound.					

resveratrol	polymer core–lipid shell	375 ± 13	–	−22 ± 1.6	76 ± 4.2	[108]
	Better stability, biocompatibility, and controlled release profile. Markedly enhanced antiproliferative activity against MCF-7 cancer cells compared to the native compound.					

resveratrol	polymer-caged liposome	212–225	0.122–0.172	4.15–14.77	74–77	[109]
	Excellent mucoadhesive characteristics and sustained drug release profiles. Stronger anti-oxidative and anti-inflammatory activity compared to the native compound.					

apigenin	monolithic	125.73 ± 5.57	0.18 ± 0.02	−26.71 ± 1.93	77.43 ± 3.62	[115]
	Exceptional colloidal stability and controlled drug release properties. Almost halved the IC_50_ value against MCF-7 and MDA-MB-231 cells.					

apigenin	monolithic	234.8 ± 12.28	0.11 ± 0.04	‒5.15 ± 0.70	55.18 ± 3.61	[116]
	Enhanced apoptosis and cell cycle arrest against HCT-116 cells. Much higher cytotoxicity compared to the native compound.					

isoliquiritigenin	polymer core–lipid shell	73.24 ± 1.83	–	−17.21 ± 0.90	96.75 ± 1.41	[120]
	Better stability in the harsh GI environment. Superior therapeutic efficacy against BC in both in vitro and in vivo studies. Higher absorption and 3.8-fold increased oral bioavailability.					

isoliquiritigenin	polymer core–lipid shell	137.2 ± 2.6	–	−34.21 ± 1.23	90.8 ± 1.5	[121]
	Modification of PLHNPs with iRGD significantly enhances their cellular internalization and antiproliferative activity against MDA-MB-231 cells. Significantly enhanced tumor-growth inhibition potential in a 4T1-bearing mouse xenograft.					

hydroxycamptothecin	polymer core–lipid shell	249.8 ± 24.4	0.289	−25.6 ± 3.8	65.93 ± 0.52	[126]
	Surface functionalization and PEG-conjugation did not modulate the drug release profiles. Ligand decoration with RGD-peptide significantly improved the cellular internalization potential and antiproliferative activity against MDA-MB-435s cells.					

hydroxycamptothecin	polymer core–lipid shell	226.4	0.236	–	–	[127]
	Much higher dose- and time-dependent cytotoxicity against both MCF-7 and HepG2 cells. 3-fold increased bioavailability after intravenous administration in SD rats compared to native compounds. Excellent therapeutic efficacy without severe side effects in murine LLC-GFP-luc lung cancer-bearing Kunming mice.					

psoralen	polymer core–lipid shell	93.44 ± 2.39	0.257 ± 0.02	−27.63 ± 0.31	76.9	[133]
	Excellent pharmaceutical attributes with controlled drug release characteristics. Higher tumor inhibition rate in MCF-7 tumor-bearing BALB/c female mice than native compound.					

ursolic acid	polymer core–lipid shell	145.1 ± 2.6	0.1 ± 0.01	−42 ± 1.2	–	[138]
	Excellent serum stability and long-term stability. Much higher cellular internalization and cytotoxicity against AsPC-1 and BxPC-3 cells.					

ursolic acid	polymer-caged liposome	135.4 ± 0.636	<0.3	+7.8	94.3	[139]
	Better stability and controlled release characteristics. Much higher accumulation in tumor and tumor growth inhibition in U14 tumor-bearing female CD-1 mice.					

baicalin	monolithic	184.3	0.177	−19.8	90.12	[144]
	Stronger cytotoxic potential against HCT-116 cells than free drug. Rapid absorption and almost 2-fold improved bioavailability after oral intake in male Albino rats.					

silymarin	polymer core–lipid shell	286.5 ± 23.8	0.23 ± 0.008	45.3 ± 8.9	97.05 ± 0.01	[149]
	14.38-fold increased bioavailability on oral ingestion in male Wistar rats. Marked decrease in blood lipid concentrations, enhanced liver function, and diminished lipid buildup in the livers of transgenic mice with NAFLD.					

piperine	monolithic	151.2 ± 4.12	0.213 ± 0.02	+24.31 ± 2.41	83.54 ± 2.88	[154]
	Excellent stability in GI fluids and much higher mucoadhesive strength. Higher cytotoxicity against MCF-7 and MDA-MB-231 cells. 6.02 times higher intestinal permeation and 4.55 times enhanced oral bioavailability.					

berberine	core–shell type hollow	149.6 ± 5.1	–	−26.8 ± 0.9	89.5 ± 4.6	[160]
	Excellent stability in GI fluids and biphasic release pattern. Significantly higher intestinal permeation and 3.4 times higher oral bioavailability.					

hesperidin	monolithic	91.43	0.056	15.6	92.8	[164]
	Better stability and controlled release characteristics. Better anti-oxidant activity.					

hesperidin	polymer core–lipid shell	314.7 ± 3.15	0.218 ± 0.05	+26.80 ± 2.21	80.18 ± 7.77	[165]
	More than two times higher skin permeation. Much better wound healing activity with a significantly higher reduction in wound size after topical application.					

emodin	polymer core–lipid shell	122.7± 1.79	0.223± 0.07	−28.5 ± 1.32	73.8	[171]
	Significantly increased cellular uptake and more than twofold decrease in the IC_50_ value against MCF-7 cells. Increased tumor inhibition rate in mice with tumors.					

#### Curcumin

Curcumin (CUR) is a yellow, bioactive compound derived from the rhizome of the plant *Curcuma longa*, commonly known as turmeric [76]. This polyphenolic substance has been traditionally used in Ayurvedic and Chinese medicine because of its numerous health benefits. CUR is known for a wide range of therapeutic effects, which include anti-inflammatory, antioxidant, antimicrobial, anticancer, cardioprotective, neuroprotective, anti-diabetic, and anti-arthritic properties [77–78]. Despite the excellent proven therapeutic efficacy, the clinical use of CUR is limited because of its complex physicochemical characteristics. CUR belongs to the “Biopharmaceutical Classification System” (BCS) class-IV drugs, representing poor solubility and permeability [79]. CUR shows very poor solubility in water because it is highly lipophilic. After, oral administration, CUR shows very limited dissolution in GI fluids and low absorption from the intestine, which results in low oral bioavailability. CUR metabolizes rapidly in the biological system, which further lowers its bioavailability, and shows a short half-life. Additionally, CUR is photosensitive and has limited chemical stability during manufacturing and storage [80–81]. To overcome these limitations, Kumar et al. developed CUR-encapsulated PLHNPs and evaluated their anticancer activity against MCF-7 cells [82]. The developed CUR-PLHNPs showed excellent pharmaceutical attributes with small particle size (PS), high zeta potential (ZP), and high entrapment efficiency (EE). The CUR-PLHNPs were tested for hemocompatibility in the drug concentration range of 60–12000 μg/mL, and the results indicated that the CUR-PLHNPs were non-hemolytic. Cell cycle analysis and apoptotic studies by flow cytometry revealed much higher apoptotic activity and cellular internalization of CUR-PLHNPs in MCF-7 cells compared to free CUR. Further, the CUR-PLHNPs showed a significantly lower IC_50_ value (i.e., 7 µg/mL) than free CUR (10.72 µg/mL). Zhao et al. fabricated RGD ligand-directed PLHNPs for site-specific delivery of CUR to treat melanoma [83]. The CUR-RGD-PLHNPs showed small PS, low polydispersity index (PDI), high ZP, and high EE. The CUR-RGD-PLHNPs showed 4.3 times higher cellular internalization in HUVECs cells than the native counterpart. Apoptotic assay and MTT assay suggested that the CUR-RGD-PLHNPs exhibited significantly higher anticancer therapeutic potential against B16 cells than the free drug. Further, CUR-RGD-PLHNPs showed much higher in vivo antitumor efficacy against B16 tumor-bearing female BALB/c mice on intraperitoneal injection for 21 days than free CUR.

#### Quercetin

Quercetin (QCT) is a flavonoid found in many fruits, vegetables, leaves, seeds, and grains. It is particularly abundant in onions, apples, berries, tea, and red wine [84]. This natural polyphenolic compound has gained attention for its diverse therapeutic effects. QCT exhibits significant anti-inflammatory, antioxidant, antiviral, anticancer, cardioprotective, neuroprotective, and anti-allergic properties [85–86]. QCT belongs to the BCS class-II drugs and shows very poor solubility. The low solubility of quercetin in aqueous environments leads to poor dissolution and absorption in the GIT, which significantly limits its bioavailability [87–88]. Moreover, QCT undergoes extensive phase-II metabolism in the liver and intestines, resulting in rapid clearance from the body and further reducing its systemic availability [89–90]. To surmount these issues, Das and co-workers fabricated QCT-loaded PLHNPs (QCT-PLHNPs) for improved transfollicular delivery to treat alopecia [91]. The developed QCT-PLHNPs provided a lipophilic environment that effectively adheres to the scalp and shows significantly higher follicular uptake. In vivo studies in Sprague Dawley (SD) rats bearing testosterone-induced alopecia suggested that QCT-PLHNPs exhibited significantly higher regrowth of hairs than free QCT. Yin and co-workers developed QCT-encapsulated cholate-modified PLHNPs (QCT-cPLHNPs) to achieve better therapeutic efficacy against leukemia [92]. Cell culture studies suggested that the QCT-cPLHNPs represented higher cell uptake and antiproliferative activity against P388 cells than pure QCT. A pharmacokinetic study in SD rats suggested that the encapsulation of QCT in cPLHNPs improves the oral bioavailability of QCT by 3.75 times compared to pure QCT suspension. Further, an in vivo therapeutic activity was conducted in DBA/2 mice bearing P388 cells-induced ascitic leukemia. The results suggested that the QCT-cPLHNPs showed superior therapeutic efficacy and represented a better survival rate compared to non-functionalized QCT-PLHNPs and free QCT.

#### Thymoquinone

Thymoquinone (THQ) is a bioactive compound derived from the seeds of *Nigella sativa*, commonly known as black cumin or black seed. This plant is part of the Ranunculaceae family and has been used for centuries in traditional medicine for its therapeutic properties [93]. THQ exhibits a wide range of therapeutic effects, including anti-inflammatory, antioxidant, anticancer, antimicrobial, antidiabetic, and hepatoprotective activities [94–95]. THQ is a BCS class-II drug, characterized by its highly lipophilic characteristics with very poor water solubility [96]. Because of the high lipophilicity, THQ shows very poor dissolution and intestinal absorption after oral administration, which results in very limited oral bioavailability. Further, THQ is sensitive to light and pH, shows poor stability in biological fluids, is extensively metabolized in the liver, rapidly eliminated from the body, and shows a very short biological half-life [97–99]. In this context, Imam et al. fabricated THQ-encapsulated chitosan–phospholipid hybrid nanoparticles (THQ-PLHNPs) for enhanced oral bioavailability and therapeutic efficacy against BC [100]. The fabricated PLHNPs showed outstanding mucoadhesive properties and stability in GI environments. Because of the excellent pharmaceutical attributes and mucoadhesive characteristics, the THQ-PLHNPs show controlled release characteristics and manyfold enhanced intestinal permeation compared to free THQ suspension. Cell culture experiments suggested that the fabricated THQ-PLHNPs significantly reduced the IC_50_ value against different BC cells. When administered orally to Wistar rats, the THQ-PLHNPs revealed a 4.74-fold increase in bioavailability compared to pure THQ suspension. Trivedi et al. investigated the therapeutic potential of THQ-PLHNPs-based gel to achieve better therapeutic activity against BC [101]. The developed THQ-PLHNPs gel showed much higher skin permeation and retention compared to the conventional THQ gel. The THQ-PLHNPs were non-irritant when applied to the skin. Furthermore, the developed gels showed higher antiproliferative activity against MCF-7 and MBD-MB-231 cells than pure THQ gels.

#### Resveratrol

Resveratrol (RVT) is a natural polyphenolic compound found in various plants, particularly in red grapes, berries, and peanuts [102]. It is known for its numerous therapeutic effects. RVT exhibits significant antioxidant, anti-inflammatory, anticancer, cardioprotective, neuroprotective, and anti-aging properties [103–104]. RVT belongs to the BCS class-II drugs with poor aqueous solubility. It shows very poor dissolution potential in GI media and very low intestinal absorption after oral administration [105]. Further, RVT extensively metabolizes before reaching the systemic circulation and is rapidly eliminated from the body. Therefore, RVT exhibits a very short plasma half-life and very low oral bioavailability These factors significantly reduce its therapeutic efficacy [106–107]. In a study, Kumar et al. fabricated RVT-encapsulated core–shell-type PLHNPs (RVT-PLHNPs) for improved therapeutic delivery [108]. The developed RVT-PLHNPs were stable for a month and showed a controlled drug release. Biocompatibility and cytotoxicity of RVT-PLHNPs were examined by using Vero cells and MCF-7 cells. The results indicate excellent biocompatibility and a higher cytotoxicity of RVT-PLHNPs than that of the free drug. Jøraholmen and co-workers fabricated polymer-coated liposomes using CHS for better topical delivery of RVT to treat vaginal inflammation and infections [109]. The developed RVT-PLHNPs showed excellent mucoadhesive characteristics and sustained drug release. The radical scavenging capability of the developed formulation was comparable to that of standard antioxidants such as vitamins C and E. The antioxidant capabilities of RVT and the developed PLHNPs were further assessed by determining improved superoxide dismutase (SOD) activities in lipopolysaccharide-induced J774A.1 cells. In vitro anti-inflammatory effects were evaluated by measuring nitric oxide (NO), tumor necrosis factor (TNF)-α, and interleukin (IL)-1β production in the same cells. The RVT-PLHNPs exhibited stronger antioxidant and anti-inflammatory activities than the free RVT solution.

#### Apigenin

Apigenin (APN) is a natural flavonoid commonly found in a variety of fruits, vegetables, and herbs. Notable sources include parsley, celery, chamomile, and oranges [110]. This compound is recognized for its wide range of therapeutic effects. APN exhibits significant anti-inflammatory, antioxidant, anticancer, neuroprotective, and cardioprotective properties [111–112]. APN belongs to the BCS class-II drugs with poor aqueous solubility. It shows very poor dissolution potential in GI media and represents very low intestinal absorption after oral administration, which limits its clinical application [113–114]. Kazmi et al. developed APN-PLHNPs for improved therapeutic effects against BC after oral intake [115]. APN-PLHNPs showed excellent colloidal stability and biphasic release profiles (initially fast and sustained afterward) over 48 h. The results from both DPPH and ABTS assays demonstrated a significant enhancement in antioxidant activity. The APN-PLHNPs nearly halved the IC_50_ value against MCF-7 and MDA-MB-231 cells after 72 h compared with the free drug. Alfaleh et al. fabricated APN-PLHNPs for improved therapeutic efficacy against colorectal cancer [116]. The therapeutic efficacy of developed APN-PLHNPs against HCT-116 cells, in terms of apoptosis and cell cycle arrest, was assessed using flow cytometry. Comparison with APN suspension showed significant improvements favoring APN-PLHNPs. Additionally, the anticancer mechanism was further explored by examining the gene expression of key signaling molecules involved in carcinogenic pathways, including Bcl-2, BAX, NF-κB, and mTOR, revealing significant outcomes for APN-PLHNPs. Overall, encapsulating APN in PLHNPs represents a promising approach to enhancing the therapeutic efficacy of APN against various diseases.

#### Isoliquiritigenin

Isoliquiritigenin (IQN) is a natural chalcone flavonoid found in various plants, particularly in the licorice root *Glycyrrhiza glabra* [117]. It is known for its wide range of therapeutic effects. IQN exhibits significant anti-inflammatory, antioxidant, anticancer, antiviral, and hepatoprotective properties [118]. As per the BCS classification, IQN belongs to class II. It shows limited solubility in the GI fluids and very low bioavailability after oral administration [119]. Wang et al. fabricated IQN-encapsulated zein phosphatidylcholine hybrid nanoparticles (IQN-PLHNPs) for improved oral efficacy against triple-negative breast cancer (TNBC) [120]. IQN-PLHNPs exhibited high stability in the harsh GI milieu. Cell culture experiment suggested that the IQN-PLHNPs showed a nearly twofold increase in cell uptake in MDA-MB-231 cells thereby better cytotoxicity was achieved. IQN-PLHNPs are effectively absorbed from the GI tract and showed a 3.8-fold increase in oral bioavailability compared to free IQN in Balb/c nude mice. The anticancer activity of IQN-PLHNPs was evaluated in MDA-MB-231 xenograft mice. The results suggested the superior therapeutic efficacy of IQN-PLHNPs. Gao and co-workers designed IQN-encapsulated iRGD-modified PLHNPs (i.e., IQN-iRGD-PLHNPs) to enhance anti-BC activity by active tumor targeting [121]. Coumarin-6 was tagged with the nanocarrier to investigate the cellular uptake potential. The developed targeted PLHNPs represented markedly enhanced cell uptake in MDA-MB-231 due to integrin receptor-mediated endocytosis. IQN-iRGD-PLHNPs also exhibited much higher cytotoxicity than non-targeted IQN-PLHNPs and free IQN. Interestingly, after leveraging iRGD peptides for active tumor-tissue accumulation and employing a stealth nanostructure for prolonged in vivo circulation, ISL-iRGD NPs exhibited superior therapeutic efficiency in mouse models bearing 4T1 breast tumors.

#### Hydroxycamptothecin

Hydroxycamptothecin (HCT) is a natural alkaloid derived from the bark and stem of the Chinese tree *Camptotheca acuminata*, also known as the “happy tree” [122]. It is part of the camptothecin family and is renowned for its potent antitumor activity. HCT exhibits significant therapeutic effects, primarily in oncology. It functions as a topoisomerase-I inhibitor, interfering with DNA replication and transcription in cancer cells, leading to cell cycle arrest and apoptosis [123–124]. However, poor aqueous solubility, instability at physiological pH, and rapid metabolism hinder its clinical utilization [125]. Yang et al. fabricated HCT-encapsulated cyclic RGD peptide (c(RGDyk))-modified PLHNPs for improved therapeutic efficacy against BC [126]. Surface functionalization and PEG conjugation did not modulate the drug release profiles. The cell culture studies indicated that the targeted PLHNPs showed much greater cell uptake in MDA-MB-435s cells by integrin receptor-medicated endocytosis than the non-targeted PLHNPs. The targeted PLHNPs showed a significant reduction in IC_50_ value (by ≈50%) compared to the non-targeted PLHNPs against MDA-MB-435s cells. Further, the targeted PLHNPs also showed better cytotoxic effects against MCF-7 cells. In another study, Ma et al. fabricated HCT-loaded PEGylated PLHNPs and optimized them by the “Quality by Design” approaches such as Plackett–Burman and Box–Behnken designs [127]. The developed nanoparticles showed excellent serum stability and revealed sustained drug release characteristics. Cell culture experiments suggested that the developed PLHNPs exhibited a much higher dose- and time-dependent cytotoxicity against both MCF-7 and HepG2 cells. After intravenous administration in SD rats, the developed PLHNPs yielded a threefold increase in bioavailability in comparison to the free drug suspension. Further, the in vivo anti-tumor activity was determined in murine LLC-GFP-luc lung cancer-bearing Kunming mice. The developed PLHNPs showed excellent therapeutic efficacy without severe side effects. Overall, encapsulating HCT into the PLHNPs can overcome the challenges related to the physicochemical properties of phytochemicals and improve drug delivery.

#### Psoralen

Psoralen is a naturally occurring organic compound found in several plants, including *Psoralea corylifolia* (Babchi), figs, celery, and parsley. It is part of the furocoumarin family and has been used for its therapeutic properties in traditional medicine [128–129]. It shows strong therapeutic potential against psoriasis, vitiligo, and leukoderma. Further, it also showed immense anticancer, anti-inflammatory, and anti-osteoporotic potential [130–131]. The clinical application of psoralen is hampered because of its poor solubility, rapid metabolism, and phototoxicity [132]. Du et al. developed psoralen-encapsulated PLHNPs for improved therapeutic efficacy against BC [133]. The developed psoralen-encapsulated PLHNPs represented controlled drug release characteristics for up to 48 h. In vivo therapeutic efficacy was determined in MCF-7 tumor-bearing BALB/c female mice. The developed PLHNPs revealed much better tumor inhibition potential than the native compound. Altogether, developing psoralen-encapsulated PLHNPs significantly improved the preclinical therapeutic efficacy and safety.

#### Ursolic acid

Ursolic acid (UA) is a natural pentacyclic triterpenoid found in various plants, including apples, cranberries, rosemary, thyme, and holy basil. It is known for its wide range of therapeutic effects. UA exhibits significant anti-inflammatory, antioxidant, anticancer, antimicrobial, hepatoprotective, cardioprotective, and anti-obesity properties [134–135]. UA is categorized as a BCS class-IV compound exhibiting low solubility and low permeability. Further, it shows low GI absorption, low bioavailability, fast metabolism and clearance, and a short biological half-life [136–137]. Markowski et al. reported the design and fabrication of UA-loaded PLHNPs for improved therapeutic efficacy against pancreatic ductal adenocarcinoma cells [138]. The formulated nanocarrier showed better serum stability and long-term stability. Cell culture studies in AsPC-1 and BxPC-3 cells suggested that the fabricated UA-PLHNPs exhibited much higher cellular internalization and cytotoxicity. Further, the developed AU-PLHNPs revealed negligible erythrocyte hemolytic activity. Similarly, Wang et al. fabricated UA-encapsulated chitosan-coated liposomes for improved antitumor efficacy against cervical cancer [139]. The formulation showed better stability and sustained release properties. Antitumor activity in U14 tumor-bearing female CD-1 mice revealed that the UA-PLHNPs exhibited much higher tumor growth inhibition. Further, UA-PLHNPs represented better tumor accumulation in vivo in comparison with the free drug.

#### Baicalin

Baicalin is a flavone glycoside derived from the roots of *Scutellaria baicalensis* (Chinese skullcap), a plant widely used in traditional Chinese medicine [140]. Baicalin shows a range of therapeutic effects, including anti-inflammatory, antioxidant, anticancer, antiviral, neuroprotective, and hepatoprotective properties [141]. Baicalin represents only limited water solubility (91 µg/mL) and 2.2% absolute oral bioavailability [142–143]. Riadi et al. fabricated baicalin-encapsulated PLHNPs to improve oral efficacy against colon cancer [144]. The developed PLHNPs represent better stability and controlled release characteristics. In vitro cell culture study revealed that the PLHNPs showed stronger cytotoxic potential against HCT-116 cells than free drug. The developed PLHNPs showed rapid absorption and almost twofold increased bioavailability after oral ingestion in male Albino rats compared to native baicalin suspension. These findings highlight the potential of PLHNPs systems in improving the therapeutic potential of poorly soluble compounds like baicalin.

#### Silymarin

Silymarin is a flavonoid complex extracted from the seeds of *Silybum marianum*, commonly known as milk thistle. Silymarin exhibits a range of therapeutic effects, notably its hepatoprotective, antioxidant, anti-inflammatory, anticancer, and cardioprotective properties [145–146]. However, the limited solubility and bioavailability of silymarin restrict its effectiveness [147–148]. To overcome these challenges and to improve the oral bioavailability of silymarin, Liang et al. fabricated silymarin-loaded PLHNPs [149]. The developed PLHNPs exhibited 14.38-fold increased oral bioavailability after oral ingestion in male Wistar rats compared to the free silymarin suspension. The hepatoprotective and antihyperlipidemic activity of the developed PLHNPs was evaluated in NAFLD-bearing PNPLA3 I148M transgenic mice. The developed PLHNPs showed a significant reduction in blood lipid levels, enhanced liver function, and decreased accumulation of lipids in the livers of mice. The PLHNPs showed hepatoprotective effects after oral administration as per the results obtained from histopathological examination of liver tissue. These findings underscore the promise of PLHNP systems in enhancing the therapeutic efficacy of phytochemicals like silymarin.

#### Piperine

Piperine (PRN) is an alkaloid found in black pepper (*Piper nigrum*) and long pepper (*Piper longum*). It is responsible for the pungency of these spices and is known for its numerous therapeutic effects. PRN exhibits significant anti-inflammatory, antioxidant, antimicrobial, antitumor, and neuroprotective properties [150–151]. However, the therapeutic use of PRN is greatly restricted because of its extremely low oral bioavailability. This poor bioavailability is attributed to its high lipophilicity and very limited water solubility (40 μg/mL) [152–153]. In this context, Kazmi et al. investigated the therapeutic potential of CHS-based mucoadhesive PLHNPs in improving PRN’s intestinal permeation, oral bioavailability, and anti-BC activity [154]. The developed PLHNPs were optimized by the “Quality by Design” approach. The optimized PRN-PLHNPs were stable in the GI milieu and showed excellent mucoadhesive strength and biphasic drug release profiles. Additionally, PRN-LPHNPs demonstrated anti-BC activity against both MDA-MB-231 and MCF-7 cells superior to that of pure PRN. An ex vivo study suggested a 6.02-fold increased intestinal permeation compared to pure PRN suspension. A 4.55-fold increased oral bioavailability was achieved after oral ingestion in Wistar rats.

#### Berberine

Berberine (BRN) is an isoquinoline quaternary alkaloid derived from various plants, including Berberis species (such as *Berberis vulgaris*), *Coptis chinensis* (Chinese goldthread), *Hydrastis canadensis* (goldenseal), and *Phellodendron amurense*. It is widely used in traditional Chinese and Ayurvedic medicine. BRN exhibits a range of therapeutic effects, including antimicrobial, anti-inflammatory, antioxidant, antidiabetic, lipid-lowering, and anticancer properties [155–157]. Despite the advantages of BRN, its clinical application is hindered by several limitations, most notably its poor water solubility and limited absorption in the GIT. It has very low bioavailability (approximately 5%) and is classified as a BCS class-IV drug. Furthermore, BRN is susceptible to the first-pass effect, which further contributes to its low bioavailability [158–159]. Yu et al. fabricated BRN-PLHNPs for improved GI stability, intestinal permeation, and oral bioavailability [160]. The developed BRN-PLHNPs were stable in GI fluids and revealed a biphasic release pattern, characterized by an initial burst release of BRN during the early phase, followed by a sustained release of BRN in the later phase, extending up to 24 h. The developed BRN-PLHNPs revealed significantly higher intestinal permeation and 3.4-fold increased bioavailability compared to the conventional BRN suspension. The improved intestinal permeation and oral bioavailability are attributed to (i) the enhanced dissolution and higher absorption due to their small PS, (ii) the modified lipophilicity, improved solubility, and controlled release of BRN, and (iii) the BRN-PLHNPs effectively overcoming the first-pass metabolism effectively.

#### Hesperidin

Hesperidin (HPN) is a bioflavonoid predominantly found in citrus fruits such as oranges, lemons, limes, and grapefruits, particularly in the peel and membranous parts of the fruits. Hesperidin exhibits significant anti-inflammatory, antioxidant, anticancer, antihypertensive, and lipid level-lowering properties [161–162]. However, its application is significantly limited by low bioavailability and poor water solubility [163]. To circumvent this, Jangde et al. fabricated HPN-PLHNPs for better topical delivery and improved antioxidant activity [164]. The HPN-PLHNPs were optimized by response surface methodology, and the optimized HPN-PLHNPs exhibited small PS, low PDI, high ZP, and high EE. The developed HPN-PLHNPs were stable for a longer period and revealed a sustained drug release profile. Further, the encapsulation of HPN in PLHNPs resulted in better anti-oxidant activity. In another study, Elmoghayer et al. fabricated HPN-encapsulated CHS-coated PLHNPs for topical treatment of wound healing [165]. The developed HPN-cPLHNPs exhibited excellent stability at refrigerator temperatures and rheological characteristics. The HPN-cPLHNPs showed significantly higher skin permeation with a 2.12 ± 0.52 times higher enhancement ratio than that of the pure HPN dispersion. The developed HPN-cPLHNPs gels showed much better wound healing activity with a significantly higher reduction in wound size after topical application for 14 days in comparison with the conventional HPN gel.

#### Emodin

Emodin (EDN) is a natural anthraquinone compound found in various plants, including rhubarb (*Rheum palmatum*), Japanese knotweed (*Polygonum cuspidatum*), buckthorn (*Rhamnus frangula*), and aloe vera (*Aloe barbadensis*). It has been used in traditional Chinese and Ayurvedic medicine for its wide range of therapeutic effects. EDN exhibits significant anti-inflammatory, antioxidant, anticancer, antimicrobial, and laxative properties [166–167]. EDN exhibits very low oral bioavailability (<3%); therefore, its clinical application is limited [168]. After administration, emodin is rapidly metabolized in the intestine by the phase-II metabolism process, forming its glucuronide conjugate, catalyzed by UDP-glucuronosyltransferases, and the parent compound becomes nearly undetectable in vivo. This process is a critical factor responsible for the low bioavailability [169–170]. To circumvent these problems, Liu et al. developed EDN-PLHNPs for enhanced therapeutic effects against BC [171]. EDN-PLHNPs revealed much higher cytotoxicity (>2 times higher) against MCF-7 cells by enhancing the cellular uptake of EDN and promoting the apoptosis of MCF-7 cells. Besides the observed morphological changes in apoptotic cells, there was a significant increase in the Bax/Bcl-2 ratio, suggesting that EDN-PLHNPs induce apoptosis in MCF-7 cells, thereby achieving an anticancer effect. Furthermore, the developed EDN-PLHNPs exhibited a notably higher tumor inhibition rate than the pure drug suspension.

### PLHNPs for combinatorial delivery of phytochemicals and/or synthetic drugs

Combinatorial delivery involves the co-delivery of two or/more different drugs and/or drug/phytochemicals and other therapeutic agents to achieve better clinical outcomes by targeting multiple pathways [172]. One of the significant advantages of PLHNPs is their ability to deliver multiple therapeutic agents simultaneously. The integration of polymers and lipids into a single nanoparticle (i.e., PLHNPs) offers several advantages, such as improved stability, controlled release, enhanced bioavailability, and the ability to carry multiple drugs or phytochemicals simultaneously. The combinatorial delivery approach with PLHNPs can be particularly beneficial in treating complex diseases such as cancer, where a single drug is often insufficient to achieve optimal therapeutic outcomes. Encapsulating both chemotherapeutic drugs and bioactive phytochemicals within the PLHNPs can provide synergistic effects, enhancing the overall efficacy of the treatment, and decreasing the chance of drug resistance by targeting multiple pathways [20,173–174]. Further, phytochemicals often have lower toxicity compared to synthetic drugs. Their inclusion in a combinatorial delivery system can reduce the overall dosage of toxic drugs required, minimizing adverse side effects and improving patient tolerance [175–176]. Recent advances in the combinatorial delivery of phytochemical and/or synthetic drugs with PLHNPs are discussed below and their pharmaceutical attributes with major outcomes are summarized in Table 2 at the end of this section.

**Table 2:** Summary of major findings of PLHNPs co-loaded with phytochemicals and synthetic drugs for the management of different solid tumors.

Encapsulated compounds	PLHNPs type	Pharmaceutical attributes	Cancer type	Cell line/animal model	Major outcomes	Ref.

cisplatin and curcumin	polymer core–lipid shell	PS: 118.5 ± 4.62 nm PDI: 0.15 ± 0.04 ZP: −13.7 ± 1.36 mV EE: >80%	cervical	HeLa and HUVEC cells/ BALB/c mice xenograft	Highest cytotoxicity via synergistic mechanism against both cell lines. Strongest antitumor effect in cervical cancer-bearing BALB/c mice model via a synergistic effect.	[177]
docetaxel and curcumin	polymer core–lipid shell	PS: 169.6 ± 4.6 nm PDI: 0.16 ± 0.06 ZP: −35.7 ± 1.9 mV EE: >80%	prostate	PC-3 cells/ PC-3 tumor-bearing mice xenografts	Excellent pharmaceutical attributes with controlled release profiles. Synergistic anticancer activity both in vitro and in vivo.	[178]
paclitaxel and triptolide	polymer core–lipid shell	PS: 160.1 ± 5.9 nm PDI: 0.17 ± 0.03 ZP: −30.4 ± 4.4 mV EE: >80%	lung	A549 cells/ A549 cells bearing mice xenograft	Much higher tumor accumulation and synergistic cytotoxicity against A549 cells. Highest tumor inhibition rate (77.4) due to synergistic effects in comparison with the singly drug-loaded PLHNPs.	[179]
resveratrol and curcumin	core–shell type hollow	PS: <160 nm PDI: NA ZP: >−10 mV EE: >90%	liver	HepG2 cells/ HepG2 tumor-bearing nude BALB/c xenograft	Ligand decoration significantly enhanced the cellular uptake and showed synergistic cytotoxicity against HepG2 cells. Much higher in vivo tumor accumulation and synergistic tumor inhibition effect.	[180]
methotrexate and β-carotene	monolithic	PS: 117.1 ± 4.6 nm PDI: 0.291 ± 0.11 ZP: ‒6.88 ± 0.26 mV EE: >60%	breast	MCF-7 cells/ DMBA-induced breast cancer-bearing female Wistar rats.	Much higher cellular internalization in MCF-7 cells by receptor-mediated endocytosis and synergistic cytotoxicity effect. Synergistic anti-tumor therapeutic effects in tumor-bearing female Wistar rats with insignificant potential toxicity.	[181]
doxorubicin and triptolide	core–shell type hollow	PS: 120 ± 5 nm PDI: 0.12 ± 0.04 ZP: −9.7 ± 0.8 mV EE: >60%	nonspecific	KB cells/ HCC tumor-bearing female athymic BALB/c-nu nude	Higher cellular internalization and synergistic therapeutic efficacy against KB cells with a low combination index. Much higher tumor growth inhibition due to synergistic therapeutic effect.	[182]
paclitaxel and tetrandrine	monolithic	PS: 139.5 ± 12.4 nm PDI: 0.13 ± 0.02 ZP: −17.2 ± 2.4 mV EE: >80%	ovarian	A2780 cells	Surface modification of PLHNPs with iRGD peptide significantly enhances cellular uptake and showed synergistic cytotoxicity against A2780 cells. The developed PLHNPs induced ROS production, promoted apoptosis, and specifically caused cell cycle arrest.	[183]

To exploit the advantages of combinatorial delivery with PLHNPs, Li et al. fabricated cisplatin (CPT) and CUR co-encapsulated PLHNPs for the management of cervical cancer [177]. The developed CPT/CUR-PLHNPs exhibited sustained drug release profiles and better stability. In vitro antiproliferative activity and synergistic effects were evaluated using HeLa and HUVEC cells. The CPT/CUR-PLHNPs revealed the highest cytotoxicity via a synergistic mechanism. The in vivo antitumor efficacy results, investigated using a cervical cancer-bearing BALB/c mice model, demonstrated that CPT/CUR-PLHNPs exhibited the most potent antitumor effect. A better therapeutic effect was achieved through (i) exploitation of the EPR effect of the tumor vasculature, that is, the PLHNPs can selectively deliver drugs to the tumor, and (ii) synergistic anticancer activity by different mechanisms of both drugs. Similarly, Yan et al. fabricated docetaxel (DTX) and CUR co-loaded PLHNPs for improved therapeutic activity against prostate cancer [178]. In vitro, DTX-CUR-PLHNPs demonstrated superior cytotoxicity and a synergistic effect between the two drugs in PC-3 tumor cells. In a mouse model with PC-3 tumor xenografts, these nanoparticles significantly suppressed tumor growth compared to other control groups, with no observable side effects. Liu et al. studied the therapeutic potential of paclitaxel- and triptolide co-loaded PLHNPs to achieve synergistic therapeutic efficacy against lung cancer [179]. Cell culture studies using A549 cells suggested that the PLHNPs represent higher accumulation in tumor cells in comparison with the free drugs. In addition, dual drug-encapsulated PLHNPs showed much higher cytotoxicity due to synergistic effects than the singly drug-loaded PLHNPs. In vivo therapeutic efficacy in lung tumor xenografts was conducted. The results indicated that the dual drug-loaded-PLHNPs exhibited synergistic therapeutic efficacy with the highest tumor inhibition rate (77.4%), which was significantly higher than that of the free drugs solution.

Another study investigated the therapeutic potential of RVT and CUR co-encapsulated SP94 peptide-engineered PLHNPs against hepatocellular carcinoma [180]. The developed PLHNPs revealed excellent pharmaceutical attributes and stability. In vitro cell culture experiments in HepG2 cells revealed that the targeted PLHNPs represent significantly higher cellular internalization by receptor-medicated endocytosis and synergistic cytotoxicity. The therapeutic activity of targeted PLHNPs was much higher than that of non-targeted PLHNPs and pure drugs, alone or in combination. A pharmacodynamic study was conducted in a BALB/c xenograft-bearing nude model. The targeted PLHNPs showed higher accumulation of the encapsulated drug in the tumor and the highest tumor growth inhibition compared with the non-targeted PLHNPs and pure drugs, alone or in combination. In another investigation, Jain et al. evaluated the therapeutic potential of methotrexate and β-carotene co-encapsulated fructose-engineered PLHNPs for the treatment of BC [181]. The developed PLHNPs showed a biphasic drug release profile and excellent storage stability for a long period at refrigerator temperatures. The surface-engineered PLHNPs showed much higher cellular internalization in MCF-7 cells by receptor-medicated endocytosis compared to non-targeted PLHNPs. Subsequently, the targeted PLHNPs revealed significantly higher cytotoxicity against MCF-7 cells than non-targeted PLHNPs. In addition, the developed dual-drug-loaded PLHNPs represented synergistic therapeutic efficacy. Further, the developed PLHNPs represented synergistic anti-tumor therapeutic effects in tumor-bearing female Wistar rats with insignificant potential toxicity. Altogether, PLHNPs offer a versatile platform for the combinatorial delivery of both synthetic drugs and phytochemicals, providing enhanced therapeutic efficacy, stability, and controlled release. By co-encapsulating multiple therapeutic agents, PLHNPs can target complex diseases like cancer more effectively, reducing drug resistance and minimizing toxicity through synergistic effects.

### Challenges ahead and future perspectives

While PLHNPs offer a promising strategy for enhancing the delivery and efficacy of phytochemicals, several challenges must be addressed to fully realize their potential in clinical settings. The formulation of PLHNPs involves intricate processes that require precise control over various parameters, including the ratio of polymer to lipid, particle size, and surface characteristics. Achieving consistent and reproducible production at a large scale remains a significant hurdle. This complexity can lead to batch-to-batch variability, impacting the overall quality and efficacy of the nanoparticles [184–185]. Ensuring the long-term stability of PLHNPs is critical for their practical application. These nanoparticles must remain stable under different environmental conditions, including temperature and humidity variations. Instability can lead to aggregation, changes in particle size, and loss of therapeutic efficacy. Developing formulations that maintain stability during storage and transportation is crucial for their widespread adoption [186–187]. Translating laboratory-scale formulations to large-scale manufacturing is a complex and costly process. The scalability of PLHNP production involves challenges such as maintaining uniform particle size distribution, ensuring reproducibility, and adhering to stringent regulatory standards. Efficient and cost-effective manufacturing processes must be developed to make PLHNPs commercially viable [188–189]. Regulatory approval for new drug delivery systems involves rigorous testing to ensure safety and efficacy. PLHNPs, being a relatively novel platform, face stringent regulatory scrutiny. Comprehensive toxicological studies are required to evaluate the biocompatibility and potential long-term effects of these nanoparticles. Additionally, the regulatory pathways for nanoparticle-based delivery systems are still evolving, which can pose delays in clinical translation [190]. Achieving precise targeting and uniform biodistribution of PLHNPs within the body is challenging. Although surface modification with targeting ligands can enhance specificity, ensuring that nanoparticles reach the intended site of action without off-target effects remains a significant hurdle. Variability in patient physiology can also impact the biodistribution and therapeutic efficacy of PLHNPs. Bridging the gap between preclinical studies and clinical application is a major challenge. Clinical trials are essential to validate the safety and efficacy of PLHNPs in humans. Furthermore, patient acceptance of nanoparticle-based therapies, including concerns about potential side effects and the complexity of treatment regimens, must be considered [191]. The early clinical success of polymeric nanoparticles and liposomes can be attributed to their unique advantages. This success is evident in the numerous liposomal drugs currently available on the market. Furthermore, a considerable number of liposomal formulations are progressing through clinical trials [192]. However, further research is necessary to establish the clinical potential of polymeric NPs. The widespread clinical studies on PLHNPs could overcome the limitations associated with both polymeric NPs and liposomal formulations, offering a more effective therapeutic option. Currently, there is no available clinical evidence for PLHNPs, likely because of their novelty and the limited amount of preclinical research conducted thus far. However, several patents have been granted across the globe that signify the potential of PLHNPs for better management of various diseases. Table 3 presents the recently granted patents related to LPHNPs.

**Table 3:** List of patents on PLHNPs.

Patent no.	Patent title	Application	Year	Ref.

CN115645523A	Application of polymer lipid hybrid nanoparticles as immunologic adjuvant and immunologic preparation.	To enhance immune responses by improving both humoral and cellular immunity with insignificant toxicity compared to traditional aluminum-based adjuvants.	2023	[196]
CN115671045A	Non-liver-targeting nucleic acid nano preparation and preparation method and application thereof.	These nanoparticles are designed to selectively target organs beyond the liver, thereby broadening the therapeutic potential of nucleic acid-based treatments for non-liver-specific diseases. This allows for precise regulation of genes, proteins, and other molecular targets, as well as modulation of the tumor microenvironment.	2023	[197]
CN115737823A	Nano drug delivery system for targeting immune cells.	It enhances the effectiveness of chemotherapy by specifically targeting immune cells involved in the tumor environment while reducing toxicity and side effects on normal cells.	2023	[198]
CN115737841A	Gene nano-medicament for enhancing anti-tumor immune effect of T cells and preparation method and application thereof.	It aims to make cancer treatments more effective by helping T-cells better target and kill cancer cells, and it could be used in treating different types of solid tumors.	2023	[199]
WO2022152141A3	Polymer conjugated lipid compounds and lipid nanoparticle compositions.	For better delivery of drugs or vaccines, making it applicable in areas such as gene therapy, cancer treatment, and vaccination.	2022	[200]
US2021379197A1	Dual-targeting lipid-polymer hybrid nanoparticles.	The developed nanoparticles specifically target cancer cells and tumor-associated cells, delivering treatment more precisely and potentially enhancing the effects of anticancer therapies.	2021	[201]
WO2021234548A1	Lipid-polymer compositions and methods of use.	Effectively encapsulates the bioactive compound and enhances its stability and bioavailability.	2021	[202]
US20210369631A1	A lipid-polymer hybrid nanoparticle.	These nanoparticles improve drug encapsulation efficiency, and stability, and provide controlled, sustained release, making them suitable for delivering therapeutic agents. The invention is versatile for both topical and systemic applications, offering better skin penetration, reduced systemic absorption, and lower toxicity.	2021	[203]
CN110960509A	Baicalin polymer lipid nanoparticle and preparation method and application thereof.	The developed nanoparticles demonstrate significant potential in treating breast cancer by inhibiting tumor growth, as tested in breast cancer cell lines and animal models.	2020	[204]
WO2019135715A1	Lipid-polymer hybrid nanoparticles.	The developed PLHNPs enhance the stability, solubility, and bioavailability of therapeutic compounds, and improve drug release profiles. The developed PLHNPs are particularly effective in treating diseases that require controlled or localized drug delivery, such as cancer, infectious diseases, or neurological disorders.	2019	[205]

Despite the abovementioned challenges, the future of PLHNPs in phytochemical delivery looks promising. Several strategies and advancements can pave the way for their successful clinical translation and widespread use. Continued research into advanced formulation techniques can enhance the stability, scalability, and reproducibility of PLHNPs. The development of personalized medicine approaches can optimize the use of PLHNPs based on individual patient characteristics. Tailoring nanoparticle formulations to patient-specific factors, such as genetic profiles and disease states, can enhance therapeutic outcomes and reduce adverse effects. Integrating PLHNPs with emerging technologies like artificial intelligence (AI) and machine learning (ML) can accelerate their development and optimization. AI and ML algorithms can predict the optimal formulation parameters, identify potential toxicity issues, and streamline the design of targeted delivery systems [193–194]. The design of multifunctional PLHNPs that combine therapeutic delivery with diagnostic capabilities (i.e., theragnostics) holds significant potential. These nanoparticles can enable simultaneous drug delivery and real-time monitoring of treatment efficacy, providing valuable insights into disease progression and therapeutic response [195]. While the current focus is primarily on chronic diseases, exploring the use of PLHNPs for a broader range of therapeutic applications can expand their impact. Investigating their potential in areas such as infectious diseases, autoimmune disorders, and regenerative medicine can open new avenues for treatment. Overall, PLHNPs represent a promising frontier in the delivery of phytochemicals, offering solutions to many of the challenges associated with conventional delivery methods. Addressing the current obstacles through innovative research and collaborative efforts will pave the way for the successful clinical translation and widespread adoption of PLHNPs in biomedical applications.

## Conclusion

PLHNPs have emerged as a promising platform for delivering phytochemicals, addressing many limitations associated with conventional delivery systems. PLHNPs leverage the strengths of both polymeric and lipid-based carriers, offering enhanced encapsulation efficiency, improved stability, and controlled release properties. By optimizing the composition and structure of PLHNPs, researchers can tailor these nanoparticles to meet specific therapeutic needs, thereby maximizing the therapeutic potential of phytochemicals. Despite the significant progress in the development of PLHNPs, several challenges remain. The complexity of formulation processes, issues with long-term stability, and difficulties in large-scale manufacturing pose substantial hurdles. Additionally, regulatory and safety concerns must be meticulously addressed to ensure the successful clinical translation of PLHNPs. Achieving precise targeting and uniform biodistribution within the body remains a critical challenge, requiring further research and innovation. Future advancements in PLHNP technology are likely to be driven by interdisciplinary approaches, integrating insights from materials science, pharmacology, and nanotechnology. Advanced formulation techniques, personalized medicine approaches, and the incorporation of AI and ML for optimization can significantly enhance the development and application of PLHNPs. By expanding the therapeutic applications of these nanoparticles beyond chronic diseases to include areas such as infectious diseases and regenerative medicine, the impact of PLHNPs can be significantly broadened. Continued research and innovation are essential to unlock the full potential of PLHNPs to fully realize their potential in clinical settings.

## Data Availability

Data sharing is not applicable as no new data was generated or analyzed in this study.
